# Perceptions and Usability of PREVENTION: A Breast Cancer Risk Assessment e-Platform

**DOI:** 10.3390/jpm13050850

**Published:** 2023-05-18

**Authors:** Samar Attieh, Marika Monarque, Andrew Durand, Saima Ahmed, Bartha M. Knoppers, Jacques Simard, Carmen G. Loiselle

**Affiliations:** 1 Division of Experimental Medicine, Faculty of Medicine and Health Sciences, McGill University, Montreal, QC H4A 3J1, Canada; 2 Ingram School of Nursing, Faculty of Medicine and Health Sciences, McGill University, Montreal, QC H3A 2M7, Canada; 3 Department of Psychology, Faculty of Arts and Science, University of Montreal, Montreal, QC H2V 2S9, Canada; 4 Department of Psychology, Faculty of Human Sciences, Université du Québec à Montréal, Montreal, QC H2X 1L7, Canada; 5 Centre of Genomics and Policy, McGill University, Montréal, QC H3A 0G1, Canada; 6 Department of Molecular Medicine, Faculty of Medicine, Université Laval, Quebec City, QC G1V 0A6, Canada; 7 CHU de Québec-Université Laval Research Centre, Quebec City, QC G1V 4G2, Canada; 8 Segal Cancer Centre, Jewish General Hospital, CIUSSS Centre-Ouest, Montreal, QC H3T 1E2, Canada; 9 Department of Oncology, Faculty of Medicine and Health Sciences, McGill University, Montreal, QC H4A 3T2, Canada

**Keywords:** breast cancer, e-health, prevention, risk assessment, risk stratification, screening, e-platform usability

## Abstract

Background: The PREVENTION e-platform was developed to provide accessible and evidence-based health information tailored to different Breast Cancer (BC) risk levels. The demonstration study objectives were to (1) assess the usability and perceived impact of PREVENTION on women with assigned hypothetical BC risk levels (i.e., near population, intermediate or high) and (2) explore perceptions and recommendations for e-platform improvement. Methods: Thirty women with no history of cancer were recruited through social media, commercial centers, health clinics, and community settings in Montreal, Qc, Canada. Participants accessed e-platform content tailored to their assigned hypothetical BC risk level, and then completed study e-questionnaires including the user Mobile Application Rating Scale (uMARS), an e-platform quality scale (i.e., in terms of engagement, functionality, aesthetics, and information). A subsample (*n* = 18) was randomly selected for an individual follow-up semi-structured interview. Results: The e-platform overall quality was high, with mean M = 4.01 (out of 5) and SD = 0.50. A total of 87% (*n* = 26) agreed or strongly agreed that PREVENTION increased their knowledge and awareness of BC risk, and 80% would recommend it to others while reporting likelihood of following lifestyle recommendations to decrease their BC risk. Follow up interviews indicated that participants perceived the e-platform as a trusted source of BC information and a promising means to connect with peers. They also reported that while the e-platform was easy to navigate, improvements were needed for connectivity, visuals, and the organization of scientific resources. Conclusion: Preliminary findings support PREVENTION as a promising means to provide personalized BC information and support. Efforts are underway to further refine the platform, assess its impact in larger samples and gather feedback from BC specialists.

## 1. Introduction

Breast Cancer (BC) represents the most common type of cancer among women worldwide [1]. In Canada, BC accounts for approximately one quarter of all new cancer cases annually, with estimates of one in eight women developing BC in their lifetime and one in thirty-four dying from it [2]. The National Institute for Health Care Excellence (NICE) guideline refers to three levels of risk for developing BC: (1) near population (less than 17% lifetime risk), (2) intermediate risk (17 to 30% lifetime risk), or (3) high risk (greater than 30% lifetime risk) [3]. Decades of epidemiologic research have led to the identification of several BC risk factors [4,5,6], with genetic susceptibility classified as high risk [3]. Lifestyle factors including alcohol consumption, smoking, physical inactivity, and hormone supplements are considered modifiable risk factors [4,5,7,8]. Conversely, age, family history, breast density and genetic susceptibility are considered non-modifiable [8].

Each of the modifiable or non-modifiable risk factors explain a proportion of variability in BC risk levels, but a combination of multiple factors can increase the risk substantially [9]. Hence, a personalized evaluation to determine overall BC risk must assess both genomic and existing risk factors to best inform clinical judgment, tailored preventive strategies, and individualized screening recommendations [10,11]. For instance, women who know and understand their BC risk level may engage in more risk reduction and preventive strategies, optimize their lifestyle practices [12], make informed health choices [13], and engage in shared decision-making with healthcare professionals [14]. Current strategies to decrease a woman’s risk of developing BC include primary prevention to reduce modifiable risk factors (e.g., avoiding tobacco, maintaining normal weight, exercise, healthy diet, etc.) and clinical approaches such as genetic counselling, rophylactic surgeries and chemoprevention medications for those at high risk [15,16].

Understanding BC-specific risks and what they entail is challenging due to related informational gaps and trusted resources [17,18]. Accessible and reliable online platforms with content adapted to personal BC risk are therefore promising to address informational needs [19,20].

The LoiselleLab team has been documenting how innovative and interactive health communication applications (i.e., internet-based tools that provide personalized health-related information and support) may benefit individuals affected by cancer. As part of the PERSPECTIVE project (Personalised Risk Stratification for Prevention and Early detection of breast cancer), led by Jacques Simard and colleagues [21], a BC risk stratification and decision support e-platform was developed and tested among women with unknown personal BC risk [22]. Stemming from this work, the team developed a revised, customized, and more interactive e-platform called PREVENTION. On the PREVENTION e-platform, women can access BC-related information tailored to their individual risk level. The e-platform includes (1) personalized features such as tailored support information, (2) screening and lifestyle recommendations, (3) scientific resources, (4) medical appointment agenda, and (5) social media and support functionalities via a mock BC support Facebook group. The main objectives of this study were to (1) assess the usability and perceived impact of PREVENTION on women with assigned hypothetical BC risk levels and (2) explore perceptions and recommendations for e-platform improvement. 

## 2. Materials and Methods

### 2.1. Procedures

A mixed-method design was used as a first step and included self-report e-questionnaires and individual semi-structured phone interviews. A convenience sample of N = 30 women with no previous history of cancer who resided in Montreal, Qc, Canada, were recruited and asked to complete study materials. A subsample (*n* = 18) was subsequently randomly selected for follow-up individual phone interviews. 

Flyers containing study details and research team contact information were posted on relevant social media accounts (e.g., Quebec Breast Cancer Foundation, Young Men’s Christian Association (YMCA)) and distributed around the McGill University campus and surrounding subway stations. The research team also set up recruitment booths at various locations and events (e.g., grocery stores, shopping malls, gynecology health clinics, libraries, community centers and the Pink Tour event organised by the Quebec Breast Cancer Foundation). Interested women shared their contact information and a research assistant called them subsequently to review study details and verify eligibility. Following consent, participants were randomly assigned to a hypothetical BC risk level with a unique login ID and password to access the PREVENTION platform. The randomly assigned differing risk levels were divided into near population (*n* = 10), intermediate (*n* = 10), or high-risk (*n* = 10). Participants only accessed the information and platform content related to their assigned risk level. All participants also viewed a mock Facebook page, with fictional testimonials, questions, and posts. After viewing all relevant content, participants were prompted to complete self-report e-questionnaires using LimeSurvey, which was, at the time, a McGill University-approved online survey platform. Randomly selected participants (*n* = 18) were invited for a follow-up phone interview that lasted between 20 and 30 min and was not recorded—a research assistant typed participants’ answers after each interview question. Participants received a CAD 20 gift e-card of their choice (i.e., Best Buy, Amazon, or Starbucks) and an additional CAD 10 gift e-card if they completed the semi-structured phone interview. This study was approved and monitored by the ethics review board of the CHU de Québec-Université Laval.

### 2.2. Measures

#### 2.2.1. Quantitative Measures

Author-generated questions included sociodemographics, level of comfort using digital technology from 1 (not at all) to 5 (very much), smartphone ownership (Yes/No), years as an Internet user, likelihood of partaking in positive health behaviors after using PREVENTION from 1 (not likely) to 5 (highly likely), overall satisfaction with the platform from 1 (very dissatisfied) to 10 (very satisfied), and essential features (Yes/No/Undecided).

The Mobile Application Rating Scale User version (uMARS) included four objective quality subscales, one subjective quality subscale, and a perceived impact section. The uMARS had good psychometric properties and internal consistency (Cronbach’s alpha; α = 0.70–0.80) on subscales with high overall consistency (α = 0.90) [23]. Each subscale included four questions, rated on a 5-point scale from 1 (inadequate) to 5 (excellent). 

uMARS four objective quality subscales:

1. The engagement subscale included five items to evaluate if the platform is entertaining, interesting, customizable, interactive, and appropriate for the target group.
2. The functionality subscale included four items to assess performance, ease-of-use, navigation, and gestural design.
3. The aesthetics subscale included three items to evaluate layout, graphics, and visual appeal.
4. The information subscale included four items to evaluate the quality and quantity of the information, the correctness of the visuals, and their credibility.

uMARS subjective quality subscale: 

The subjective quality subscale explored willingness to recommend the e-platform, willingness to pay for it, anticipated frequency of use, and overall quality. 

uMARS perceived impact:

This section assessed changes in participants’ perceived BC related awareness, knowledge, attitudes, intentions (e.g., speak to a physician), help-seeking (e.g., following recommended BC medical steps), and intention to change health behaviours (e.g., following the BC risk recommendations)

The perceived impact of the platform consisted of 6 items ranging from 1 (Strongly disagree) to 5 (Strongly agree).

#### 2.2.2. Interview Guide

An author-generated semi-structured interview guide (Table 1) was developed to explore participants’ perceptions of PREVENTION. 

### 2.3. Data Analysis

#### 2.3.1. Quantitative Data

All quantitative data were analysed using the IBM SPSS (Statistical Package for Social Science) software, version 27 (Licensed materials -Property of IBM Corporation and its licensors, USA). Frequency and descriptive statistics were used to summarize participant characteristics and additional platform questions. uMARS scores were computed by averaging the four objective subscales. The mean scores for each subscale (i.e., engagement, functionality, aesthetics, and information) were averaged to obtain the overall uMARS score for objective quality; a subjective quality subscale was calculated separately. Means and standard deviations were calculated for the uMARS objective and subjective subscales, and frequencies were calculated for perceived platform impact. A one-way multivariate analysis of variance (MANOVA) was conducted to determine if there were mean differences in the 5 uMARS subscales (engagement, functionality, aesthetics, information, and subjective quality) between risk level groups (near population, intermediate, high).

#### 2.3.2. Qualitative Data

We conducted a thematic analysis using an inductive approach and coded verbatim following steps described by Braun and Clarke [24]: familiarization with the data, generating initial codes, searching for themes, reviewing themes, defining, and (re) naming themes. Two members of the research team read the transcripts and analyzed them independently. Codes were refined, transformed into themes, and further classified. Disagreements were discussed via debriefing sessions leading to consensus [25,26]. Transparency is addressed herein by providing relevant quotes in the Section 3.

## 3. Results

### 3.1. Participant Characteristics

The sample included all women (N = 30) between 26 and 60 years old (M = 42.27, SD = 10.90). A total of 70% (*n* = 21) of participants had a university degree (i.e., Bachelor’s, Master’s, or professional degree) and 60% (*n* = 18) worked full-time. A total of 46% (*n* = 14) were married, and 53% (*n* = 16) reported having children. All participants reported being Internet users, with an average of M= 17.50 years of use (SD = 4.96). On a scale of one (not at all) to five (very much), participants felt comfortable with technology (M = 4.33, SD = 0.92) and 93% (*n* = 28) owned and had used a smartphone for anywhere between three and fifteen years. (Table 2).

### 3.2. Quantitative Results

#### 3.2.1. uMARS Objective and Subjective Quality Scale

uMars scores are reported in Table 3. The overall mean uMARS score was high, with a mean M = 4.01, SD = 0.50 (range from 3.91 to 4.16). The uMARS objective quality subscales scored highest for information M = 4.43, SD = 0.53 (range 3.0–5.0) and functionality M = 4.32, SD = 0.62 (range 2.8–5.0), and lower for aesthetics M = 3.80, SD = 0.63 (range 2.7–5.0) and engagement M = 3.49, SD = 0.72 (range 2.3–5.0). The mean for subjective quality subscale was M = 3.28, SD = 0.74, indicating a good overall quality.

A one-way MANOVA was conducted to determine whether there was a statistically significant difference between women’s risk-level groups (i.e., near-population, average, high) and their scores on the uMARS subdomains: information, functionality, aesthetics, engagement, and subjective quality. An evaluation of the homogeneity of variance-covariance matrices assumptions underlying MANOVA indicated that the assumption was met at the 0.05 level (χ^2^(df = 30) = 33.80 (*p* = 0.75). MANOVA revealed no statistically significant differences in scores on the uMARS subscales between the risk-level groups, Wilk’s lambda (0.92), F(10,46) = 0.204, *p* = 0.99, indicating that perceived platform quality and acceptance were similar regardless of assigned BC risk-level. 

#### 3.2.2. uMARS Perceived Impact

Table 4 summarizes the perceived impact of using PREVENTION. In total, 87% of participants agreed or strongly agreed that PREVENTION increased their knowledge and awareness of BC risk. Moreover, 77% agreed or strongly agreed that PREVENTION changed their attitude towards BC risk assessment, with about half (57%) reporting that it increased their motivation to talk with their physician about BC risk. About half also agreed or strongly agreed that PREVENTION would encourage them to follow the recommended medical steps for their BC risk level (57%), and 80% reported that the platform would help other women follow BC risk recommendations.

#### 3.2.3. Involvement and Satisfaction with e-PREVENTION

Table 5 and Table 6 display participant involvement and satisfaction with PREVENTION, and the features they consider essential. As a result of using PREVENTION, 80% of women reported being somewhat or highly likely to take part in their own healthcare and follow the lifestyle recommendations to decrease their risk of BC. Overall, on a scale of one to ten, participants were satisfied with PREVENTION (4.0–10.0, M = 7.77, SD = 1.36); 80% reported that they would recommend the platform to others. A total of 63% chose “personalized recommendations” as their most liked feature, and almost all participants (97%) considered this feature to be essential. As for the mock BC support Facebook group, 70% disliked this feature and 23% considered it nonessential. 

### 3.3. Qualitative Findings

**Figure 1:**
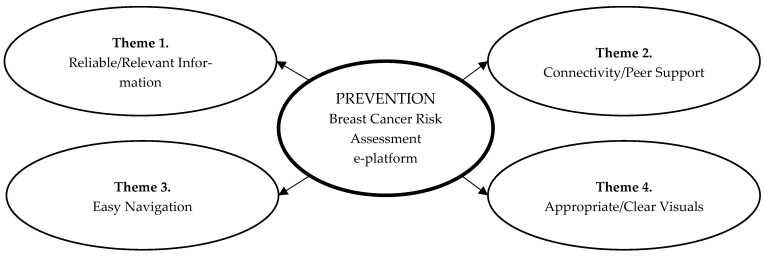
Main findings regarding the e-PREVENTION e-platform including 4 key themes.

Overall, participants perceived PREVENTION as useful, reliable, comprehensive, and informational. Four main themes were identified (Figure 1): (1) Reliable source of relevant information, (2) Promising means for connectivity and peer support, (3) Ease of navigation, and (4) Appropriateness and visual appeal. Each theme highlights PREVENTION’s perceived benefits and suggested areas for improvement. Each theme is reviewed in turn.

#### 3.3.1. Theme 1: Reliable Source of Relevant Information

Perceived benefits. Participants perceived PREVENTION as a trusted source of information. Their comments reflected the quality of the scientific information and the relevance of the content in educating women on breast cancer. For instance, women spoke highly about the scientific value, credibility, and completeness of the available information. Despite their attempts to search the Internet for health-related information, women were cautious when consulting online resources. One woman explained: “with Internet, we need to be careful. If it was a good source, I would feel more confident with the information” (translated from French; ID: gbzt6QTC). Similarly, another participant shared the need for trusted and credible sources of information: “I am cautious with which websites I consult […] The source needs to be reputable” (ID: qd6im7k). Moreover, women perceived the information on PREVENTION to be helpful, important, and essential—with the right balance between science and accessibility. One woman stated, “Well, I thought it was very useful […] Also, there were many things I didn’t know. The information wasn’t too much, just the most important […] The information was essential but short” (ID: Wxfw2jCm).

The health recommendations on PREVENTION helped women learn about specific risk factors (e.g., genetic risk), stratified BC risk levels, available resources, screening suggestions, and how to manage BC risk. Women mostly identified the “recommendations” section as the feature that stood out. For example, one participant stated:

“The screening and understanding risk. That helped me. I had a little idea of mammogram, the section on it helped me understand more. The ages listed were helpful. The clinical breast exam was good to know what exactly it was. I also liked the “at risk” and how age and family history play a role. Lifestyle was so interesting, like how much I drink and my weight. Just great information. I think people have a general idea, but this actually helped with the specifics.”

(ID: xxm5f3n).

More specifically, participants liked the BC “follow-up and screening recommendations” and considered them useful, thought-provoking, and an important aspect of PREVENTION. Most participants reported that they would likely follow these recommendations. One woman said, “I started getting screenings years ago, but I stopped […]. With the information on your website [PREVENTION] I changed my mind because the risk is there.” (Translated from French; ID: erw87HQC). Another woman stated, “It would make anyone want to follow-up after reading it. You just want to be sure and take those steps…” (ID: 1ubqkhm). Furthermore, several participants reported the importance of having access to scientific articles and resources available in PREVENTION. Participants frequently used the Internet to find health-related information, but they did not always have access to scientific resources. When we asked participants if they would read scientific articles, one woman replied, “Yes, because sometimes there are studies done that I cannot access that you need to pay for […] I think it is very important for us to all have access to research and articles.” (ID: uyxuc8a).

Areas for improvement. Overall, participants wanted additional information that delves deeper into certain topics (e.g., breast cancer screening). One woman shared her wish for more information “on the tests, maybe even a picture of the result. For example, what is a mammogram result?” (Translated from French; ID: untydsm). More information on preventative strategies (i.e., self-examinations or mastectomies) were also requested: “Women like Angelina Jolie who don’t have breast cancer but had [a mastectomy]. So, what’s the deal? Is it good to have? Would having both breasts removed lower my mastectomy breast cancer risk? If so, what does it involve?” (ID:uyxuc8a).

Some participants wanted more details on BC development and risk. A participant suggested adding “[…] a section on prevalence depending on ethnicity, since I think some groups are more susceptible than others” [translated from French; ID: untydsm]. She also would have liked to know more about “[…] the risk of cancer related to the environment”. Another participant wanted to know about “[…] the odds [of having BC if you do not have a family history], compared to someone who has a family history” (ID: 1ubqkhm). Although PREVENTION was designed to focus on BC prevention, participants also would have welcomed information on what to expect after a breast cancer diagnosis. Such comments included:

- “Personally, I would like to know more about screening and the diagnosis. More when we have breast cancer, what are the steps to take.” (Translated from French; ID: qd6im7k);
- “You touched everything lightly. In terms of treatment protocol, like what are the stages of breast cancer? What are the treatments?” (ID: xxm5f3n);
- “More on surgeries. I know there are different types, like breast removal and lump removal. I want to know more.” (ID: uyxuc8a).

#### 3.3.2. Theme 2: A Promising Means for Connectivity and Peer Support

Perceived benefits. PREVENTION promoted a sense of connectedness (i.e., referring to individuals interacting with peers). Some participants welcomed the idea of a Facebook feature. One participant mentioned that it helped them feel less alone: “Sometimes I feel alone, and it made me feel less alone, especially when I saw the Facebook page” (ID: 1ubqkhm). Other participants voiced the importance of social media for women with high BC risk or those with a current cancer diagnosis. For example, when asked if they would want to connect and share with others, one woman said, “it’s always good to share information. If I was a patient especially” (ID: wuntufs). Participants (*n* = 10) preferred reading other people’s online stories and experiences but preferred not to share or post themselves. For example, one woman said, “I would [go on the Facebook group] to read, but not share” (translated from French; ID: erw87HQC), and another said, “I would read and not post” (ID: epmnt97).

Areas for improvement. Participants suggested that the connectivity (i.e., between individuals and technical features of the platform) of Facebook and the Agenda needs refinement. Some participants (*n* = 10) felt that they did not need a Facebook feature. Some were not interested in interacting with peers on Facebook, and others found it intrusive. They preferred this feature to be integrated within the e-platform. One participant explained, “I think instead of a private group on Facebook, it would be better on the platform. This way people can interact with each other and not know their names. I would feel more comfortable.” (ID: x89pedm) Another participant reported, “Well, the comment about the Facebook page, like just a link… Maybe it would be interesting to integrate a discussion board on the site.” (ID: z7qg6af). 

Likewise, several participants disliked the agenda feature and felt its usability was limited since it did not connect to other software (e.g., Gmail, iCalendar, etc.). The lack of connectivity prevented some participants from using the feature altogether. Participants recommended connecting the reminders on the agenda feature with their personal devices, calendar, or emails. Two participants shared suggestions on how to improve this feature:

“I didn’t like the agenda feature. I think it should be linked to a Gmail account so that you don’t have to go to the site to see if we have an appointment. I already have enough going on; I wouldn’t use it.”

(ID: epmnt97).

“If you’re going to have [the agenda feature] on there, it should have an alarm connected to it that will pop-up like a Google calendar or iPhone calendar. When you put it in, you can select when it reminds you. Other than that, I don’t think it’s really necessary because most people just use their phones.”

(ID: xxm5f3n).

#### 3.3.3. Theme 3: Ease of Navigation

Perceived benefits. Most participants (*n* = 17 out of 18) agreed that going through PREVENTION was easy and straightforward. They found the navigation bar easy to use and intuitive, and the sections flowed easily one into the other with well-ordered topics. One woman said “I thought the sequence was very smart and well defined. It followed a good rhythm […]” (ID: 1ubqkhm), and another stated “It’s a drop-down menu and you just click, and it opens. It’s not a long list. Just easy.” (ID: uyxuc8a). Women were able to find the information they needed easily: “We don’t have to look, everything is well placed together” (translated from French; ID: pr0kxv4); “If I had a question, straight away, I found an answer.” (Translated from French; ID: erw87HQC). 

Areas for improvement. Participants requested improvements related to the organization of the scientific resources. Some participants found this section overwhelming, with too many outbound links. They suggested making it more concise and accessible. Two participants explained:

- “The literature section was overwhelming. Maybe edit it down to 4–5 of the most important or comprehensive Canadian references” (ID: mp86fhi);
- “The literature section needs to be cleaned up. More sub-categories and blurbs on each site would help, with hyperlinks rather than direct URL links. It would be more appealing and look cleaner. The information there is good, just needs better organization/look.” (ID: 8ufavpv).

#### 3.3.4. Theme 4: Appropriate and Clear Visuals

Perceived benefits. Participants were in favor of the platform’s design. The colors were appealing to most participants, and the website had a “clean and professional” look. PREVENTION’s design matched our objectives and target population, with one woman saying “It’s good for a scientific website that wants to inform people. It’s good. It looks serious” (translated from French; ID: untydsm). Several participants also commented on the appropriateness of the colors, especially the pink, which they associated with breast cancer. For example, one woman said “The pink color is for breast cancer, that was important to see” (ID: 1ubqkhm). The pictures were appropriate, interesting, and helped capture participants’ attention. One woman commented that “[…] The pictures were very inclusive; the first picture has women of different ages and races. I liked the pictures” (ID: Wxfw2jCm).

Areas for improvement. Some participants suggested adding specific pictures or more colours, adjusting the layout, and improving the readability of PREVENTION. The pictures on PREVENTION could have been more engaging and less “standard”. For example, a woman stated “The pictures used are very obviously stock photos, I think it’s a bit boring. I feel like anyone could go on Shutterstock and find them. I would like to see more relevant pictures, more personal touches” (ID: 8ufavpv). Although the colors were generally liked, certain participants either wanted more variety or more vibrant/warm colours that still maintained PREVENTION’s balance and scientific impression. Two participants explained: “I liked the colors. I find the look is overall very clean. I am someone who likes color a lot. It could have more colour, more in the heading or background to feel warmer. The white is a bit stale […] The white made me feel like I was reading journal articles online” (ID: mp86fhi), “I found it very basic. I am not a designer, but it was basic. Basic but functional. I would like more colors.” (ID: Wxfw2jCm).

A few participants recommended that the platform become more dynamic or interactive by adding graphs to help illustrate the content. One woman suggested adding videos or animated figures by saying: “Without putting too much, but [I recommend] a place with videos. To be more dynamic. Maybe someone that explains. Animated figures would be fun” (translated from French; ID: pr0kxv4). A few women also commented on the layout and design, such as large empty spaces. One woman voiced that “Some sections have images which creates big blanks, looks bad. I think you could change the layout, perhaps be more centered, then there would be no blanks” (ID: 8ufavpv). Some participants also found the text too small, dim, and suggested adding larger and clearer fonts to improve readability. Two shared opinions: “The font could be larger. There is a lot of information, with the small font, it’s hard to read” (ID: wuntufs); “[…] the colour of the lettering was a bit dim to read” (translated from French; ID: pr0kxv4).

## 4. Discussion

In this demonstration study, we assigned hypothetical BC risk levels to 30 women and assessed their perceptions regarding PREVENTION, an informational e-platform tailored to their individual BC risk levels. Findings from uMARS indicated overall high-quality ratings, with higher scores for content and functionality compared to aesthetic and engagement domains. Study findings also showed that PREVENTION has the potential to promote behavioral changes. For instance, participants reported a high likelihood of further engaging in healthcare and preventive behaviors (e.g., lifestyle changes, seeking medical support) to help lower their own BC risk after consulting the PREVENTION platform. Consistent with our findings, Afshin, Babalola [27] reviewed 224 studies and found that Internet and mobile interventions could help improve lifestyle behaviors and reduce risk factors for non-communicable diseases. Similarly, a meta-analytic review by Roberts, Fisher [28] found that online health platforms helped increase physical activity uptake in cancer survivors. A few e-tools exist to estimate BC risk, but none provide trusted resources for individually tailored, risk-related information post risk assessment [29]. Similar to our objective of creating an e-platform to address important informational gaps, an Australian team [20] developed a prototype called iPrevent for clinicians and patients to asses BC risk collaboratively. Tailored risk management information was also provided based on the Australian National Guidelines. iPrevent demonstrated good usability, with possible areas for improvement. PREVENTION was developed within the Canadian context, informed by best practices and extant evidence. Taken together with the present findings, online informational platforms such as PREVENTION could help fill informational gaps, motivate end-users to engage proactively in their health and understand screening options, and help individuals to engage in shared decision making with their healthcare providers. An accurate understanding of BC risk levels and what each entails is fundamental to motivate behavioral changes and adherence to medical interventions [30]. Unfortunately, the Internet may lack high quality, evidence-based health information [31,32]. Thus, it appears plausible that the perceived trustworthiness of PREVENTION increased women’s willingness to adhere to health recommendations. 

Participants herein only accessed the information pertinent to their assigned hypothetical BC risk level. There were no statistically significant differences between how women from different risk levels perceived PREVENTION. Tentatively, this indicates that perceived platform quality is similar across BC risk levels; however, the small sample size limits conclusions that can be drawn here. Furthermore, during interviews, women requested more BC information including on breast cancer screening and preventive strategies. Certain women preferred accessing all relevant BC information, regardless of whether it was personally pertinent to them, in a ‘’more is better’’ approach. These findings align with individual preferences for cancer information whereby *intense seekers* seek as much information as possible (e.g., scientific reports and professional websites) to fulfill their information needs [33].

Beyond significant support for PREVENTION’s content and functionality, its aesthetics and perceived engagement need enhancement. Women reported, for instance, the need to improve design elements and feature implementation and address issues in platform engagement. PREVENTION could benefit from more interactive layouts, warmer colors, and less generic images. The lower engagement ratings could also be explained by PREVENTION’s designed features not yet being fully developed (e.g., social media mock Facebook, agenda feature, scientific resources). For instance, one of the key principles for effective e-platforms is to design with usability, readability, and navigability in mind [34,35]. User-centered design, for example, leverages design principles to create platforms that are easily usable and understandable by end-users, which can foster engagement. Using elements of codesign, which entails developing e-health applications in conjunction with end-users from the very start, could also impact engagement. Including personalized elements, professionalism, shaping (i.e., keeping demands on the user low), visual cues, and a variety of features may also increase engagement [36]. Hence, PREVENTION can be improved by having a better design, including an interactive layout and better feature synchronization. 

Similarly, the social media feature is another area in need of improvement. Whereas some participants were hesitant to trust the social media content and challenged its quality, others enjoyed interacting with others and liked sharing stories. While peers on social media can create supportive and empowering groups, concerns still exist surrounding confidentiality and reliability of information posted [37]. Indeed, evidence gaps still exist regarding the effective use of social media for health purposes [38]. Developing PREVENTION’s social media feature will therefore require careful consideration of the emerging evidence and end-users’ preferences. Suggestions to include a forum option directly linked to the PREVENTION platform can be explored to address privacy concerns and potential intrusions. 

This study has a few limitations. First, semi-structured interviews were not recorded and only transcribed by the research assistant conducting the interviews; this might translate into less accuracy in reporting participants’ opinions. Second, participants were assigned a hypothetical BC risk level when accessing PREVENTION. This precludes inferring how women with an actual formally identified risk level, or BC experience would respond to PREVENTION. Third, some content features of PREVENTION were not fully developed, which might have impacted perceived usability by participants. For example, the social media features were developed using mock pages and social media posts to give participants an idea of how these features may work in the future. Similarly, the agenda feature was not fully integrated into the platform. Caution is warranted when interpreting the usability and participants’ interest in features that are not yet fully functional. Last, the study sample was small with 30 participants completing e-questionnaires and a subset (i.e., 18) taking part in interviews. Despite these limitations, the use of a mixed methods approach relying on quantitative and qualitative data collection provided a complementary and rich process to gather participant perceptions.

## 5. Conclusions

This demonstration project aimed to begin to assess the usability and perceived impact of PREVENTION among women with assigned hypothetical BC risk levels and explore their perceptions and recommendations for platform improvement. Preliminary findings indicated that PREVENTION is of high quality overall. Its most appreciated features included access to timely BC risk knowledge, awareness of available evidence, and tips for proactive behavioral changes. As the incidence of BC continues to rise, e-platforms such as PREVENTION present accessible, cost-effective, trusted and sustainable options to address BC informational gaps and support for health-related decision-making. Next steps include platform revisions based on participant feedback, testing with larger sample sizes, and exploring further BC specialist perceptions and recommendations. Identifying women’s true BC risk levels prior to having them engage with PREVENTION will be key to add to the evidence in support of the platform. 

## Figures and Tables

**Table 1:** Semi-structured individual interview questions.

Question Type	Question(s)
General	What was your first impression of the platform?
	We have chosen certain pictures and colors for the platform. Did you like them? Would you suggest something else?
	Did you find it easy to navigate through the various sections of the platform?
	What information stood out to you?
	Did the order of the topics make sense?
	What did you like about the platform?
	What did you not like about the platform?
Feature Specific: Recommendations	The platform includes recommendations for follow-up activities based on risk levels. Is this something you are interested in? Why? Why not?
Feature Specific: Resources	Do you search online for answers on your health questions?
	If websites and specific articles were recommended on the platform, would you read them?
	Are there any specific topics related to breast cancer that you want to know more about? If so, which ones?
Feature Specific: Share/social media	Would you be interested in connecting with other people online to discuss your breast cancer risk?
	Would you go on a private Facebook group and share your thoughts and ask questions? Or would you prefer to read and not post?
Knowledge	Did you find the content of the platform useful?
	Did you learn new things? If yes, please describe.
Comments	Is there anything you would like to see added or changed? This can be about the topics and information, the way it looks or even the way it works.
	Do you have any more suggestions, comments, or questions?

**Table 2:** Participant characteristics (N = 30).

	Min	Max	M (SD)	Median
Age	26	60	42.27 (10.90)	40
Technological skills				
Comfort with Technology (from 1 to 5)	2	5	4.33 (0.92)	5
Using the Internet (years)	10	35	17.50 (4.96)	18
Using a Smart Phone (years)	3	15	7.78 (2.95)	5
	*n*	%		
Owns a Smart Phone				
Yes	28	93.3		
No	2	6.7		
Education				
High school or equivalent	4	13.3		
Pre-university (vocational) degree	5	16.7		
Bachelor’s degree	12	40		
Professional degree (e.g., medical, law)	1	3.3		
Master’s degree	8	26.7		
Marital Status				
Married/common law	14	46.7		
Single (never legally married)	12	40		
Separated/divorced	4	13.3		
Has Children				
Yes	16	53.3		
No	14	46.7		
Work Status				
Full time	18	60		
Part time	3	10		
Self-employed	1	3.3		
Unemployed	2	6.7		
Homemaker/stay at home parent	2	6.7		
Retired—not due to health issues	2	6.7		
Student	2	6.7		
Has Social Support				
Yes	25	83.3		
No	5	16.7		

**Table 3:** uMARS overall and domain-specific mean scores by breast cancer (BC) risk levels.

	Hypothetical BC Risk Levels											
	Near Population (*n* = 10)			Intermediate (*n* = 10)			High (*n* = 10)			Total (N = 30)		
Subscales	Min	Max	M (SD)	Min	Max	M (SD)	Min	Max	M (SD)	Min	Max	M (SD)
Engagement	2.5	4.8	3.35 (0.69)	2.3	5.0	3.65 (0.73)	2.3	4.8	3.48 (0.79)	2.3	5.0	3.49 (0.72)
Functionality	2.8	5.0	4.28 (0.73)	3.8	5.0	4.45 (0.54)	3.5	5.0	4.23 (0.62)	2.8	5.0	4.32 (0.62)
Aesthetics	2.7	4.3	3.73 (0.49)	3.0	5.0	3.97 (0.60)	2.7	5.0	3.70 (0.79)	2.7	5.0	3.80 (0.63)
Information	3.0	5.0	4.30 (0.55)	3.8	5.0	4.58 (0.47)	3.5	5.0	4.43 (0.57)	3.0	5.0	4.43 (0.53)
Overall uMARS scores			3.91 (0.49)			4.16 (0.43)			3.96 (0.59)			4.01 (0.50)

**Table 4:** Percentages of perceived impact after using e-PREVENTION (N = 30).

	Strongly Disagree	Disagree	Neither Agree/Disagree	Agree	Strongly Agree
	*n* (%)	*n* (%)	*n* (%)	*n* (%)	*n* (%)
Increase BC awareness	0 (0)	2 (6.70)	2 (6.7)	16 (53.3)	10 (33.3)
Increase BC knowledge	0 (0)	2 (6.70)	2 (6.7)	17 (56.7)	9 (30.0)
Increase BC attitudes	1 (3.3)	1 (3.3)	5 (16.7)	20 (66.7)	3 (10.0)
Intention to change ^a^	1 (3.3)	2 (6.70)	10 (33.3)	13 (43.3)	4 (13.3)
Help seeking ^b^	1 (3.3)	2 (6.7)	10 (33.3)	13 (43.3)	4 (13.3)
Behaviour change ^c^	0 (0)	3 (10.0)	3 (10.0)	21 (70.0)	3 (10.0)

^a^ Increased their motivation to talk to a physician about BC risk; ^b^ encouraged to follow recommended medical steps for their BC level, if needed; ^c^ likely follow BC risk recommendations.

**Table 5:** Participants’ perceived involvement and satisfaction after using e-PREVENTION (N = 30).

	Not Likely	Somewhat Unlikely	Neither Likely or Unlikely	Somewhat Likely	Highly Likely
	*n* (%)	*n* (%)	*n* (%)	*n* (%)	*n* (%)
Engage in healthcare ^a^	0 (0)	0 (0)	5 (16.7)	15 (50)	9 (30)
Use email reminders	2 (6.67)	3 (10.00)	3 (10.00)	11 (36.67)	11 (33.67)
Screening/app.	1 (3.33)	0 (0)	3 (10.00)	15 (50.0)	11 (36.67)
Follow lifestyle recommendations	0 (0)	2 (6.67)	4 (13.33)	12 (40.00)	12 (40.00)
				*n*	*%*
Device used to access PREVENTION	Desktop			27	90.00
	Tablet			1	3.33
	Smartphone			2	6.67
Would Recommend PREVENTION	Yes			24	80.00
	No			6	20.00
Favorite Feature	Recommendations			19	63.33
	Resources			10	33.33
	Facebook Group			1	3.33
Least Favorite Feature	Recommendations			2	6.67
	Resources			7	23.33
	Facebook Group			21	70.00

^a^ One missing data entry.

**Table 6:** Essential e-PREVENTION features.

Essential Feature	Yes *n* (%)	No *n* (%)	Undecided *n* (%)
Personal recommendations	29 (96.7)	1 (3.3)	0 (0)
Screening recommendations	28 (93.3)	2 (6.7)	0 (0)
Lifestyle recommendations	27 (90.0)	1 (3.3)	2 (6.67)
Scientific Articles	26 (86.7)	3 (10.0)	1 (3.3)
Facebook Group	20 (66.7)	7 (23.3)	3 (10.0)
Agenda Feature	25 (83.3)	2 (6.67)	3 (10.0)
FAQ Feature	27 (90.0)	0 (0)	3 (10.0)
Links to other Websites	22 (73.3)	7 (23.3)	1 (3.3)
References	21 (70.0)	5 (16.7)	4 (13.3)

## Data Availability

The datasets generated during and/or analyzed during the current study are not publicly available at this time but are available from the corresponding author upon reasonable timeline request.
